# Linkage of Medicare insurance claims to police-reported motor vehicle crashes: advancing traffic safety research in older adult populations

**DOI:** 10.1186/s40621-026-00658-5

**Published:** 2026-02-06

**Authors:** Fang-Wen Lu, Andrew R. Zullo, Allison E. Curry, Melissa R. Pfeiffer, Marzan A. Khan, Nina R. Joyce

**Affiliations:** 1 Department of Epidemiology, Brown University School of Public Health, Providence, USA; 2 Center for Gerontology and Health Care Research, Brown University School of Public Health, Providence, USA; 3 Center for Injury Research and Prevention, Children’s Hospital of Philadelphia, Philadelphia, USA; 4 Department of General Pediatrics, University of Pennsylvania Perelman School of Medicine, Philadelphia, USA

**Keywords:** Motor vehicle crashes (MVCs), Medicare, Data linkage, Older adults, New Jersey Safety and Health Outcomes (NJ-SHO), Administrative data

## Abstract

**Background:**

Motor vehicle crashes (MVCs) are a leading cause of injury among adults aged 65 years and older (“older adults”). As the number of older drivers grows, it is increasingly important to understand clinical factors associated with an increased risk of MVC. A major barrier, however, is the lack of data. To address this, we linked two large-scale administrative databases, the New Jersey Safety and Health Outcomes (NJ-SHO) Data Warehouse, which contains information on all police-reported crashes in New Jersey from 2004 to 2019, and Medicare Fee-for-Service (FFS) insurance claims, which contains health care encounters and prescription drug dispensings among older adults in the United States over the same period. This paper explains the linkage process, describes selected work leveraging these data to study MVCs in older drivers, and highlights features and strengths of this linkage for future research.

**Methods:**

The NJ-SHO–Medicare linkage was performed using categories of name (first and last), sex, age (birth and death date), and residence (state and ZIP code). Matches were ranked by quality and overall confidence.

**Results:**

After comparing different match strategies, we accepted a match when (1) the name match quality was High or Medium and the age match was High or (2) the name, sex, and residence match categories were all High. Of the 2,722,773 individuals successfully linked, we accepted 2,661,782 matches (97.76% of individuals linked and 91.59% of those submitted for linkage). All accepted matches were Strong or Fair. Among accepted matches who enrolled in Medicare FFS in 2019, 342,422 (28.57%) were 65–69 years old, 619,437 (51.69%) were female, and 955,309 (79.72%) were non-Hispanic White. Only 29,561 (2.47%) experienced an MVC and 25,478 (2.13%) received a citation. The most prevalent clinical conditions ever diagnosed were cataracts (669,044; 55.83%); chronic pain, fatigue, and fibromyalgia (367,165; 30.64%); and glaucoma (287,420; 23.98%).

**Conclusions:**

With extensive temporal and population coverage, the NJ-SHO–Medicare linkage supports studying the relationships between clinical exposures (e.g., medications ), driving events (e.g., crashes, citations) and medical care trajectories, which can help advance the driving safety of older adults and inform future efforts to integrate administrative data.

**Supplementary Information:**

The online version contains supplementary material available at 10.1186/s40621-026-00658-5.

## Background

Motor vehicle crashes (MVCs) are a leading cause of injury among adults aged 65 years and older (“older adults”). In 2022, approximately 26 older adults were killed and over a thousand were injured in an MVC in the United States each day [1]. By 2050, it is expected that the number of older drivers will almost double, with older adults composing 25% of the licensed population in the United States [2]. Thus, identifying risk factors for and developing interventions to prevent crashes and crash-related-injuries have become top priorities for health care professionals and organizations that serve older adults.

A major challenge to studying causes of MVCs among older adults is the lack of data. An MVC is a relatively rare event and thus it can be prohibitively resource intensive to collect data on enough older adults for a long enough period at the level of granularity necessary to generate precise estimates. While several large administrative crash databases contain information on select subsets of all crashes, such as fatal crashes (e.g., the Fatality Accident Reporting System [FARS]) or crashes that result in an auto insurance claim, these data have limited information on the characteristics of the drivers involved in the crash (“crash-involved drivers”). Conversely, while several large administrative health care databases, such as Medicare insurance claims or hospital discharge data, contain detailed information on the crash-involved driver, they are limited to crash-involved drivers who seek medical care for an injury and contain little information on the nature of the crash [3].

A handful of prospective cohort studies have collected information on both driving and clinical exposures of interest in older drivers, such as a diagnosis of dementia or the use of certain prescription drug medications. The largest and most comprehensive of these studies is the LongROAD study, which followed approximately 3,000 older adults for five years, collecting longitudinal information on their medical conditions, prescription drug use, characteristics of their driving habits (e.g., frequency, location, conditions), and recording all crashes that occurred [4]. The LongROAD study has provided invaluable insights into the risk factors for crashes among older drivers, particularly among older drivers with Alzheimer’s disease and related dementias [5]. However, with only 2,069 (69%) participants having completed the five-year study, it is still limited in its ability to estimate causal effects for less common exposures, such as the use of prescription medications or procedures (e.g., hip surgery).

In this paper we describe the development of a novel data source – Medicare insurance claims linked to driver licensing, suspension, citation, and police-reported crash data from the New Jersey Safety and Health Outcomes (NJ-SHO) Data Warehouse for over 2.6 million drivers in New Jersey over a 16-year period. By linking the comprehensive clinical information available in Medicare claims with the detailed licensing and crash information available in NJ-SHO Data Warehouse, we can overcome many of the data limitations of prior studies. The structure of this paper is as follows. We begin by describing the various Medicare and NJ-SHO datasets included in the linkage and the process by which they were linked. We then discuss the strengths of this linkage, highlighting questions that investigators are now able to address, and describing three case studies from our own work. Last, we discuss some of the remaining limitations of these data before previewing potential areas for expansion to overcome these limitations.

**Table 1:** Description of datasets and key data elements for traffic safety research used in the NJ-SHO–Medicare data linkage

**Datasets**	**Description**	**Key data elements**	**Years**
NJ-SHO			
Licensing	Data on each driver who had a valid driver’s license or permit in New Jersey during the study period	Dates for the start and end of the permit period, probationary period, and full license period, along with the respective starting ages for each period, organ donor status, postal code	2004–2019
Citations	Originally from New Jersey Administrative Office of the Courts events, with detailed records of the event nature	Date and type of the adjudicated traffic citation	2004–2019
Suspensions	Same as above, also from New Jersey Administrative Office of the Courts events	Dates of suspension and restoration, suspension type, total number of suspensions for this person	2004–2019
Crash	Detailed crash-, driver-, and vehicle-level information for police-reported crashes^*^	Crash date and impact (e.g., injuries), alcohol involvement, driver age, vehicle type, safety equipment (e.g., airbag, seat belt), contributing causes (e.g., following too closely, unsafe speed)	2004–2019
Medicare			
MBSF	Data on basic demographic details, enrollment, and a comprehensive chronic condition data warehouse	Insurance year, residence state, race, sex, birth and death dates, coverage of Part A, B, C and D during each month of the year, chronic conditions and the earliest diagnosis (e.g., Alzheimer’s disease)	1999–2022
MedPAR	Claims for Medicare Part A payment from two sources: inpatient and skilled nursing facility (SNF)	Claim from and through dates, admission source (e.g., physician referral), ICD-9/10-CM codes, procedure codes, beneficiary zip code, cost	1999–2022
Carrier	Claims for Medicare Part B billed by doctors, including claim and line-level details	Claim from and through dates, BETOS Code (e.g., lab tests, electrocardiograms), ICD-9/10-CM codes, procedure codes, cost	1999–2022
Outpatient (Part B)	Claims for Medicare Part B hospital institutional billing	Claim from and through dates, facility type (e.g., rural health clinics), ICD-9/10-CM codes, provider state, cost	1999–2022
Part D	Claims for drug-related payment	Service date, quantity (e.g., 30 tablets), supply days, drug brand and generic names, product strength (e.g., 50 mg), cost	2007–2022
OASIS/Home Health	Claims for care received at a patient’s home	Claim from and through dates, referral type (e.g., SNF transfer), claim facility type (e.g., HHA), ICD-9/10-CM codes, cost	1999–2022
MDS 2.0 and 3.0	Health screening and assessment results for long-term care nursing facilities residents	Entry date, discharge date, assessment reference date, HIPPS codes, RUG codes, assessments (e.g., hearing ability, vision, cognitive ability, mood, mobility), ICD9/10-CM codes, height/weight	1999–2022

^*^In New Jersey, crashes are reportable if they involve injury, death, or damage of $500 or more

NJ-SHO = New Jersey Safety and Health Outcomes; MBSF = Master Beneficiary Summary File; MedPAR = Medicare Provider Analysis and Review; MDS = Long Term Care Minimum Data Set; OASIS = Home Health Outcome and Assessment Information Set; BETOS = Berenson-Eggers Type of Service; ICD-9/10-CM = International Classification of Diseases, 9th/10th Revision, Clinical Modification; HHA = Home Health Agency; HIPPS = Health Insurance Prospective Payment System, RUG = Resource Utilization Group

## Methods

### Overview of the datasets

Table 1 contains detailed information on key data elements present in each of the datasets within the NJ-SHO Data Warehouse and the Medicare claims data. Here, we provide a high-level overview of the different datasets and highlight important features of the data.

1. NJ-SHO

The NJ-SHO Data Warehouse consists of multiple datasets from New Jersey, linked together at the individual level. Every individual with at least one record in any source dataset during the study period is assigned a unique ID that is common across the NJ-SHO Data Warehouse. Records from each source were combined into a single file, which was then processed with probabilistic linkage software to identify records belonging to the same person. For a detailed description of the linkage process for the datasets that constitute NJ-SHO, see Curry et al. [6]. Although the NJ-SHO Data Warehouse links numerous datasets, our work is limited to the datasets pertaining to driving and crashes, which include the licensing, events (i.e., suspension, citation), and crash datasets.

#### The licensing dataset

The licensing dataset supplied by the New Jersey Motor Vehicle Commission includes start and end dates for a permit, probationary, and full license. From this raw data we constructed a dataset that includes a record with the start and end dates of every license phase for every individual with a New Jersey driver’s license from 2004 to 2019. This allowed us to determine license status on any given day for every individual in the NJ-SHO Data Warehouse.

#### The citations dataset

The NJ-SHO Data Warehouse contains a dataset with a record for every “event” that occurs against someone’s license. We created an analytic dataset consisting of citation events only. Each record contained the reason for the citation (e.g., speeding, failure to yield) and the date and time of the adjudicated traffic citation.

#### The suspensions dataset

From the raw events dataset, we created a second analytic dataset with a record for every license suspension. Like the citation events, each record contains the reason for the suspension. The suspension records also include the suspension type (e.g., basic driving privilege, registration privilege, all privileges) and suspension and restoration dates.

#### The crash dataset

The crash dataset includes every police-reported crash in New Jersey from 2004 to 2019. A crash is reportable to police in New Jersey if it results in an injury or at least $500 in property damage. Examples of the data collection form for police-reported crashes can be found at New Jersey Department of Transportation website [7]. The crash dataset is maintained as separate datasets, each at a different level of analysis. The “crash-level” dataset contains a unique record for every crash that occurred, including the time, date, and location of the crash in addition to detailed information on the actions leading to the crash (e.g., failure to yield), road conditions, light conditions, total number of people injured, and other details. The crash-level dataset also includes the latitude and longitude for the location of the crash (for 85% of crashes), allowing investigators to calculate the distance from the crash to any other geocoded location. The “driver-level” dataset includes a unique record for each driver involved in the crash, along with information about the vehicle they were driving. Each record in the driver-level dataset also includes the corresponding unique crash-ID from the crash-level dataset. Thus, two drivers who collide will each have a unique record in the driver-level dataset that can be linked to the same crash-ID in the crash-level dataset.

2. Medicare

The Medicare program provides health insurance to all U.S. residents 65 and older as well as those younger than 65 who have a qualifying disability, end-stage renal disease, or Amyotrophic lateral sclerosis. There are two types of payment systems in Medicare: Fee-for-Service (FFS) and Medicare Advantage (sometimes referred to as “Part C”). Medicare FFS pays for each unit of service, whereas Medicare Advantage uses a capitated payment model (i.e., “bundled payment system”) involving fixed payments per enrollee to private insurers. Any care received while a beneficiary was enrolled in a Medicare Advantage plan was not included in the current linkage.

We included various Medicare FFS claims datasets spanning from 1999 to 2022, with the exception of Part D drug coverage, which only began in 2006 (prescription drugs were not covered under Medicare FFS before 2006) and has been more complete for research purpose since 2007. Future researchers can anticipate additional years of Medicare data as new datasets become available. However, for most past research, we define the eligible population as individuals enrolled from 2007 to 2019, when the datasets overlap. This overlap allows us to establish a comprehensive treatment and traffic history for these individuals. For each year that a beneficiary is alive and participating in the Medicare program, they generate an enrollment record in the Master Beneficiary Summary File (MBSF), which includes demographic information and monthly indicators of their enrollment in the different components of Medicare FFS (Parts A, B, and D) and Medicare Advantage (Part C). All records for each person have the same encrypted 15-character Medicare beneficiary ID so that data generated by the same individual can be connected across all years in which they are enrolled.

Anytime a Medicare beneficiary receives care for which the Medicare program reimburses a provider, a claim is generated. Each dataset within the Medicare data warehouse contains claims for different types of care, with substantial overlap. Because the claims are generated for reimbursement purposes, where a claim is located depends on how it was billed. This can result in the same visit generating claims in multiple datasets, and even in two claims for the same type of care (e.g., an emergency department visit) appearing in different datasets. For instance, patients receiving outpatient care from a physician based in a hospital may have claims in both Medicare Part A and Part B, with Part A paying for supplies and procedures and Part B paying for physician services [8]. In contrast, for two patients admitted to the same emergency department, one may generate a claim in Medicare Part B and the other in Medicare Part A, depending on the employer of the attending physician they see (i.e., employed by the hospital or not). Thus, investigators need to query multiple datasets to create a comprehensive picture of patient care. More information on each respective Medicare dataset can be found in Mues et al. [9] or on the Research Data Assistance Center (ResDAC) website [10].

### Structure of the linked data

Individuals were linked based on following elements: first name, last name, date of birth, date of death, sex, state, and ZIP code. The data linkage was conducted by an experienced third party, Acumen, which is a contractor of the National Institute of Aging (NIA) and supports linking data provided by Center of Medicare & Medicaid Services (CMS) for NIA grantees via the NIA’s Data Linkage Program. Throughout the linkage process described below, the study team collaborated with Acumen to define match qualities and determine which matches to include based on the information provided by Acumen. The linkage study was approved by the institutional review boards of Brown University and Children’s Hospital of Philadelphia (CHOP).

**Fig. 1:**
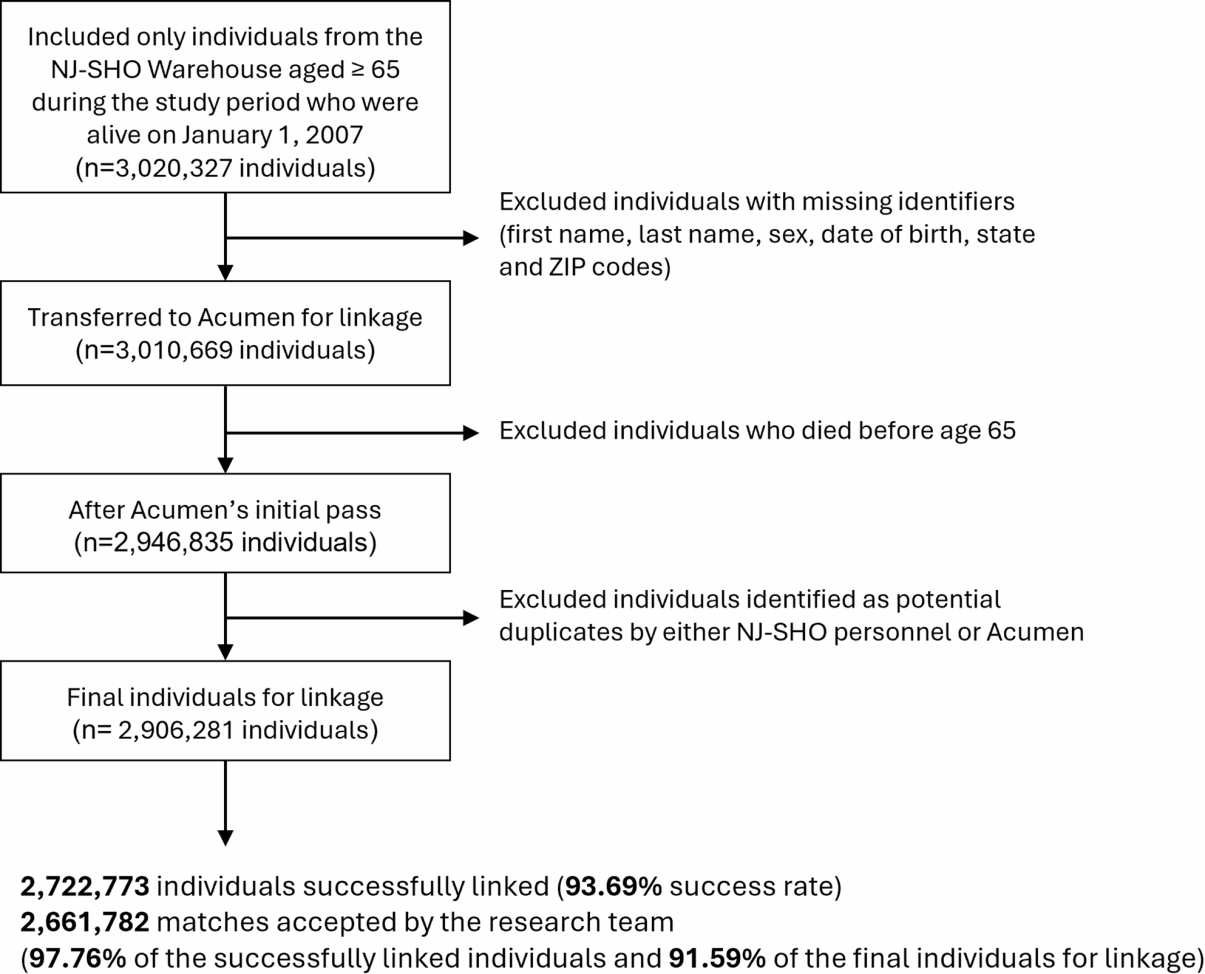
Workflow for linking individuals from New Jersey Safety and Health Outcomes (NJ-SHO) crash and licensing datasets to Medicare Fee-for-Service (FFS) records

Figure 1 summarizes the key steps involved in the data linkage process. First, study author (MRP) from the NJ-SHO Center for Integrated Data conducted data cleaning to create a finder file for Acumen. Individuals were included if they had a record in the licensing or crash dataset and if they were born from 1900 to 1954, as the goal was to include people who are eligible for Medicare due to age —meaning they need to be 65 or older during the period for which we have data (2007–2019). After excluding individuals who died in or before 2006, the population included 3,020,327 people. The second step involved restricting the dataset to those with non-missing values in first name, last name, sex, date of birth, state, and ZIP code, yielding 3,010,669 people. A new NJ-SHO ID was created for each of these individuals; their data was then transferred to Acumen for data examination and linking with the Medicare FFS claims data.

As a result of investigation by Acumen and preliminary linkage exploration, we identified and excluded individuals who died before their 65th birthday, resulting in 2,946,835 individuals advancing to the next round of the linkage. The second round involved identifying potential duplicates (i.e., two NJ-SHO IDs linked to the same Medicare beneficiary); after excluding the duplicates identified by either NJ-SHO or Acumen, the final cohort included in the data linkage consisted of 2,906,281 individuals.

Of those records in the final cohort, 2,722,773 individuals (93.69%) were successfully linked to a Medicare enrollment record and each candidate linkage was evaluated based on four main variable categories: name (first name and last name), sex, age (date of birth and date of death), and residence (state and ZIP code). Acumen evaluated the match quality in each variable category and assigned a rating of High, Medium, Low, or None. For example, if two records matched exactly on both first and last name, the matching category “name” was assigned a match quality of “High.” If only the last name matched, the match quality would depend on the nature of the match on first name (e.g., John vs. Jon would result in a “Medium” match quality where John vs. Frank would result in a “Low” match quality). Definitions for the category-specific match quality ratings (High/Medium/Low/None) used for name, sex, age, and residence are provided in Supplementary Table 1. Each individual was then given an overall match confidence rating of Strong, Fair, or Weak. For example, a “High” match rating for all four categories (name, sex, age, and residence) resulted in match confidence of “Strong,” whereas a “High” match on name but only a “Medium” match quality for the other categories would result in an overall match confidence of “Fair.” Supplementary Table 2 summarizes the primary patterns used to assign an overall match confidence rating of Strong, Fair, or Weak from the match quality ratings across four categories. We present 1-to-1 matches separately from resolved 1-to-many matches, which occurred when an NJ-SHO individual matched to multiple Medicare records and Acumen selected the single best linkage. The combination of match quality and match confidence allows researchers to tailor the population based on the importance of different factors. For example, since our analyses focused on older adults, we prioritized a “High” match on age and evaluated five different strategies based on variable-level match quality or overall match confidence (Table 2).

**Table 2:** Alternative match acceptance strategies evaluated

Strategy	Acceptance criteria	Accepted matches	% of final individuals for linkage (*n* = 2906281)	Key trade-offs
1	Accept Strong + Fair, reject all Weak	2,712,325	93.33%	Simplest approach, but likely too inclusive (it would accept matches where both name and age match quality were Medium or Low). Relies solely on the overall match confidence score
2	Accept matches with 3–4 Highs	2,621,889	90.21%	May be too strict in practice. It can exclude otherwise strong matches with Medium name match quality (and High match quality for sex and age) because residence match quality is Medium, even though people move
3	Name (High/Medium) AND Age High	2,588,334	89.06%	Overly restrictive on age. Requiring High match quality on age allows little room for common errors in birthdate components (day/month/year)
4	Name (High/Medium) AND Age (High/Medium)	2,589,162	89.09%	Effectively the same as Strategy 3. The small relaxation in age does not meaningfully change which matches are retained
5	[Name (High/Medium) AND Age High] OR 3 Highs in Name, Sex, and Residence	2,661,782	91.59%	Balanced and pragmatic approach: It prioritizes age match quality, while also incorporating sex and residence information and retaining matches when name, sex, and residence are all High even without High age match quality

## Results

**Fig. 2:**
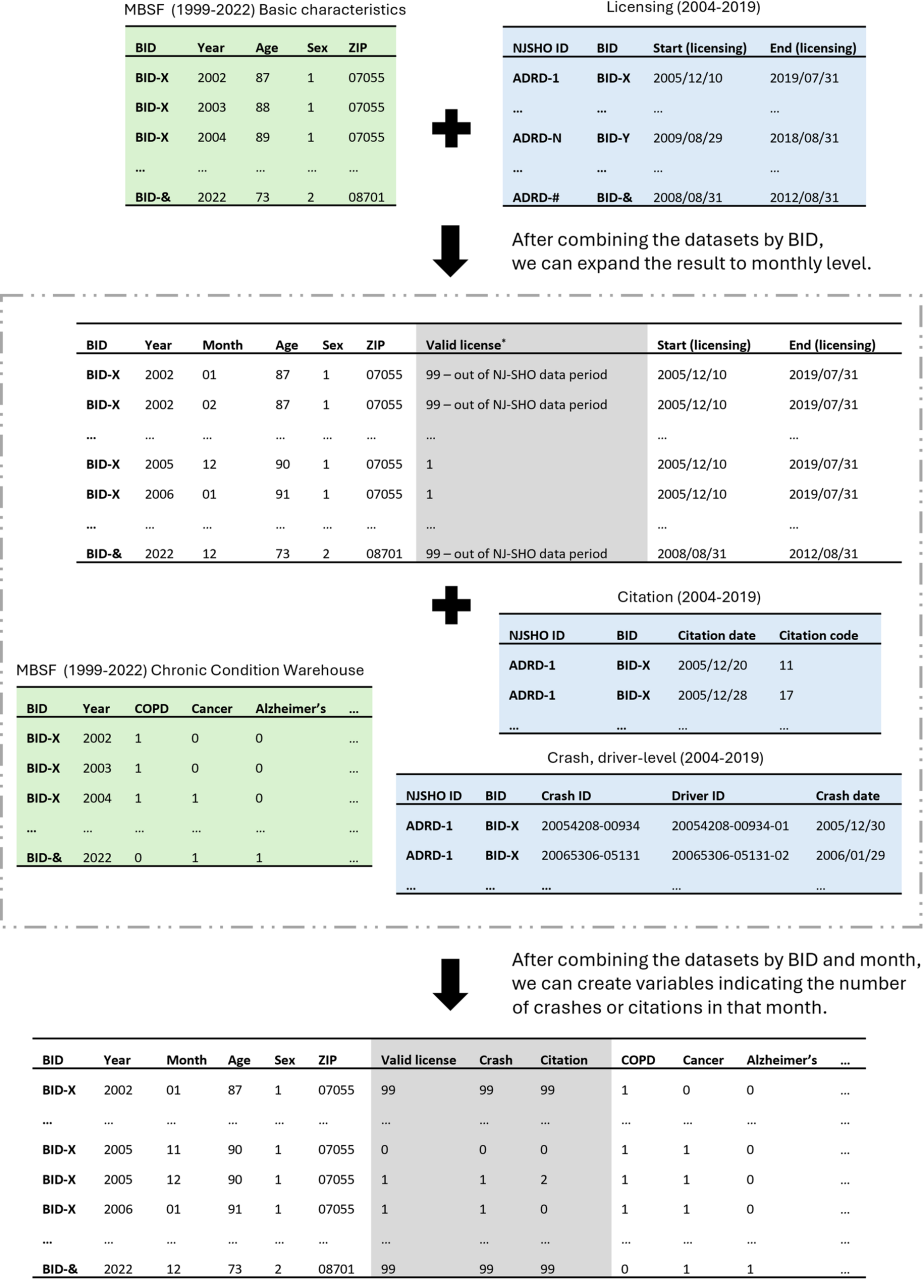
Demonstration of the data integration process combining datasets from the New Jersey Safety and Health Outcomes (NJ-SHO) Data Warehouse and Medicare claims data using synthetic datasets. Green areas represent datasets from the Medicare claims data, blue areas represent datasets from the NJ-SHO Data Warehouse, and gray areas indicate variables created during the integration process. *Denotes individuals who held a valid license on at least one day within the month

After comparing five candidate match acceptance strategies (Table 2), Strategy 5 was selected and applied to define the set of accepted linkages used in subsequent analyses. In this strategy we accepted a match when either of the following conditions was met (1) the name match quality was High or Medium and the age match was High or (2) the name, sex, and residence match categories were all High. This strategy achieved a balance between retaining matches and prioritizing match quality on age. All the accepted matches under these criteria were Strong or Fair matches (*n* = 2,661,782, 97.76% of the successfully linked individuals and 91.59% of the final individuals for linkage, see Fig. 1; Table 2). The researchers could then link the NJ-SHO and Medicare datasets described in Table 1 using a common variable assigned after linkage called BID (see Fig. 2 for examples of how these datasets were linked together and examples of what the datasets would look like after linkage).

Table 3 presents the selected characteristics of individuals based on the accepted matches who were aged 65 years or older and enrolled in Medicare FFS throughout 2019 or until death in 2019. There were 1,198,381 individuals selected, and the median (Q1–Q3) age was 69 (73–79), with most individuals in the 65–74 age range (28.57% aged 65–69 and 27.77% aged 70–74). The proportion of males and females was similar, with females accounting for 51.69% of the population. The majority were non-Hispanic White (79.72%), and 3.66% died during the year. In 2019, 2.47% of these eligible individuals had at least one crash, 2.13% received at least one citation, and 63.14% were licensed all year. In terms of chronic conditions ever diagnosed, cataracts (55.83%); chronic pain, fatigue, and fibromyalgia (30.64%); and glaucoma (23.98%) were the most prevalent. In contrast, for those not accepted (i.e., either not linked at all or linked but not accepted), only basic demographic characteristics were available: until the end of 2019 (or death), the median (Q1–Q3) age was 75 (69–84); individuals aged 65–69 accounted for 27.16% and those aged 70–74 accounted for 18.01%; and females accounted for only 43.96% of unaccepted individuals.

**Table 3:** Selected characteristics of individuals in the NJ-SHO–Medicare linkage in 2019

Characteristic	*N* (%)
**Demographics**	1,198,381
Age	
Median (Q1, Q3)	69 (73–79)
65–69	342,422 (28.57)
70–74	332,741 (27.77)
75–79	223,828 (18.68)
80–84	142,898 (11.92)
>= 85	156,492 (13.06)
Female	619,437 (51.69)
Race/Ethnicity	
American Indian/Alaskan Native	339 (0.03)
Asian/Pacific Islander	51,574 (4.30)
Black (or African American)	84,343 (7.04)
Hispanic	64,003 (5.34)
Non-Hispanic White	955,309 (79.72)
Other	13,338 (1.11)
Unknown	29,475 (2.46)
Died during the year	43,803 (3.66)
**Selected NJ-SHO data elements**	
Crashed	29,561 (2.47)
Received a citation	25,478 (2.13)
Licensed all year	756,615 (63.14)
**Selected Medicare data elements**	
Chronic conditions (ever diagnosed)	
Cataracts	669,044 (55.83)
Chronic pain, fatigue, and fibromyalgia	367,165 (30.64)
Glaucoma	287,420 (23.98)
Sensory deafness and hearing impairment	178,962 (14.93)
Alzheimer’s disease and related dementia	145,267 (12.12)
Mobility impairments	43,440 (3.62)
Epilepsy	29,369 (2.45)
Schizophrenia and other psychotic disorders	26,975 (2.25)
Spinal cord injury	14,954 (1.25)
Traumatic brain injury (TBI) & nonpsychotic mental disorders due to brain damage	13,873 (1.16)
Attention-Deficit/Hyperactivity Disorder (ADHD) and other conduct disorders	8497 (0.71)
Sensory blindness and visual impairment	6524 (0.54)
Multiple sclerosis and transverse myelitis	6234 (0.52)
Muscular dystrophy	666 (0.06)

N=Number, %=Percent, NJ-SHO=New Jersey Safety and Health Outcomes, FFS=Fee-for-Service, Q1=Lower Quartile, Q3=Upper Quartile

## Discussions

### Strengths of the NJ-SHO–Medicare linkage

A major advantage of the NJ-SHO–Medicare linkage is the ability to identify appropriate denominators when calculating the rate of MVC in a population. Most databases with information on crashes lack access to the number of licensed individuals in the target population and often use census data as the denominator instead (e.g., FARS). In contrast, the NJ-SHO data includes information on everyone with a valid license in New Jersey, which allows for more precise denominators. In terms of the linkage, the combination of NJ-SHO and Medicare provides researchers with detailed information not only on crashes but also individuals’ health records. Other data sources like FARS offer valuable data on fatal MVCs, though these incidents represent a limited subset of all incidents. Lacking comprehensive pre-crash health data and linkage to health databases, the ability to use FARS in assessing the influence of individual health status on crash outcomes is still limited. By linking detailed crash data with health claims, researchers can access reliable information and investigate a wide range of questions [11], such as treatment received for crash-related injuries and how medications patterns change before and after the crash. Additionally, because NJ-SHO also includes data on license suspensions and citations, this linkage supports broader investigations into various traffic-related outcomes beyond crashes.

In the following paragraphs, we provide three examples that demonstrate the types of questions that can be explored using this data warehouse. Note that the process of linking NJ-SHO and Medicare in the following example studies was slightly different from the process described in the current linkage paper. The current linkage adopted a more rigorous method, using algorithms to evaluate the likelihood of matches between variables with similarities, such as the names ‘John’ and ‘Jon.’ In contrast, the linkage utilized in the examples relied on exact matching between the linking variables, though it mimicked a probabilistic approach by including various versions of the name. Additionally, the population involved in each process differed; the current linkage includes more years of data.

### Examples of how these data have been used

1. Distance from home to MVC location

By linking the NJ-SHO Data Warehouse with Medicare records, researchers can have the geographical location of crashes, the geographic location of the crash-involved driver’s residence, and their health conditions at the same time. Joyce et al. [12] drew on the insights provided by this linkage to investigate the relationship between crash distance from home and drivers’ characteristics. The researchers used the Euclidean distance between a driver’s home and the crash site and assessed crash distances in relation to various chronic medical conditions. The results indicated that more than half of the crashes occurred within three miles of the driver’s home. Additionally, of all the chronic conditions examined, drivers diagnosed with sensory blindness and visual impairments had the greatest differences in crash distances compared to those without these conditions. Although the study was descriptive in nature, the findings provided real-world evidence that might inform policy discussions on driving license restrictions for older adults. This linkage enabled a deeper exploration of how medical conditions might be associated with driving behavior, and the combination of geographical and medical information allows future researchers to explore broader hypotheses.

2. Non-benzodiazepine hypnotics and MVC risk

With Medicare data, researchers can not only understand what medical conditions a driver has, but also what procedures clinicians performed and what drugs were prescribed and dispensed (Medicare Part D). Using information about drug dispensing, as well as linked licensing and crash records, Zullo et al. [13] conducted a study examining older adults’ use of non-benzodiazepine hypnotics (“Z-drugs”), often prescribed for insomnia, and the risk of MVCs. The study adopted a sequential target trial emulation approach, aiming to estimate the effect of Z-drug initiation and subsequent sustained use on the risk of MVCs during a 12-week follow-up for each emulated trial. The results indicated no significant differences in MVC risk following Z-drug initiation; however, sustained Z-drug use may have reduced MVC risk. The extensive information on crash time and type also allowed the researchers to conduct stability tests, further examining whether the effect differed between at-fault and not-at-fault crashes, as well as between pre-midday and post-midday crashes. The study demonstrates the observational data linkage’s usefulness in answering questions for causal inference that would otherwise be impossible to address through randomized controlled trials.

3. Differences in driver characteristics associated with crash and crash-related injury

By combining police crash reports with claims, the NJ-SHO–Medicare linkage facilitates a more comprehensive understanding of crashes by distinguishing between crash events and crash-related injuries. Joyce et al. [3] conducted an exploratory descriptive analysis leveraging the advantages of this linkage and identified differences between crashes and crash-related injuries across various factors such as demographics, clinical conditions, and prescription drug use. Recognizing these distinctions is crucial for researchers in making valid causal inferences and aiding policy-making decisions. The study emphasized the importance of researchers being aware of whether their outcome of interest is a crash or a crash-related injury, as the differences in group characteristics may suggest different mechanisms and necessary covariate adjustments in their models.

### Limitations

Although the linkage opens new avenues for future studies, some limitations are worth noting, as they may limit the conclusions that can be drawn. First, although this data linkage is quite comprehensive, it does not include information on the amount of time spent driving (“driving exposure”). Without information on driving exposure, it is not possible to determine if differences in crash risk are due to differences in the amount of time someone spends driving and thus is at risk of crashing. Notably, the linkage still provides some insight into driving exposure, as it allows researchers to identify older adults who no longer hold a valid driver’s license, thereby helping to address this limitation to some degree. Second, the licensure and traffic-related event data from the NJ-SHO Data Warehouse are limited to the state of New Jersey, which may have a specific driving landscape and particular regulations and lack the capability to capture crashes involving New Jersey drivers that occur outside the state. Therefore, conclusions drawn from this data warehouse may have limited generalizability when making inferences about driver behavior in other states. Third, the healthcare data used in this linkage, Medicare, suffers from some widely discussed limitations associated with using claims in research for determining health status, such as possible disease misclassification and lack of risk factor and disease severity information if it is not related to reimbursement [9]. Fourth, the current linkage did not include Medicare Advantage records, even though the proportion of older adults enrolled in Medicare Advantage plans has been increasing. In 2019, Medicare Advantage plan enrollees represented over 37% of the total Medicare population [14]. As a result, linking only Medicare FFS individuals may lead to a substantial loss of information about the population. Additionally, we only linked police-reported MVCs with Medicare FFS claims; consequently, crashes where drivers do not call the police may go unrecorded. Previous research using three data sources indicated that relying only on hospital and police reports may underestimate the total number of crashes [15]. Also, we were unable to examine the rate of false positive matches due to the lack of gold standard, the resource-intensive nature of the manual review process and the sensitivity of the individual data involved, which makes obtaining permission nearly impossible. However, since all of our included matches are classified as strong (81%) or fair (19%), we believe the percentage of false positives should be relatively small. For those interested in conducting data linkage in the future, Acumen’s most recent linkage procedure can be downloaded from the NIA website [16]. This updated procedure uses a match quality band ranging from 1 to 7, instead of the High/Medium/Low/None ratings that were used when we conducted this linkage. We did not adopt the updated procedure because it was released after linkage was completed, which took more than six months due to iterative review between study authors and Acumen staff to evaluate linkage quality. Given the similarity in the overall linkage framework, the updated procedure is unlikely to substantially change our results.

### Area for expansion

As the NJ-SHO–Medicare linkage serves as a valuable data source, there are several opportunities to further expand and enhance this type of linkage to deepen our understanding of the relationship between driving and health. For example, NJ-SHO data could be linked with Medicaid and Medicare Advantage to improve the representativeness of the older adult population in the U.S. Additionally, for states participating in all-payer claims databases (APCDs), linking both public and private insurance records could provide a better understanding of the health status and driving characteristics of the working-age population. While the current data linkage is limited to drivers in New Jersey, each state has its own unique driving patterns and demographics. To improve the wellbeing of road users, it is crucial to utilize the data available in each state. We hope that this paper will benefit researchers interested in using the NJ-SHO–Medicare linkage and serve as a starting point for future researchers to explore the possibilities of linking traffic and health data. We are open to sharing more details of our experiences with colleagues from other states or countries to facilitate similar efforts.

## Conclusions

The NJ-SHO–Medicare linkage established a large retrospective cohort that enables researchers to examine the relationships between driving events (e.g., crashes and citations) and medical trajectories in older adults. Covering at least sixteen years and more than two million individuals, this linkage makes it possible to study changes in health characteristics and care trajectories before and after driving events and provide valuable information that can help inform strategies for enhancing road safety among older drivers in the United States. Furthermore, by demonstrating the power of large-scale administrative data linkages, this work serves as a precedent for future efforts to integrate disparate data sources and advance injury prevention research.

## Supplementary Information

Supplementary Material 1.

## Data Availability

Data dictionaries and supporting documentation (e.g., analytic code) are publicly available on GitHub (http://doi.org/10.5281/zenodo.17429852). Use of data from the Centers for Medicare & Medicaid Services (CMS) was covered under the strict terms of a Data Use Agreement (DUA) and individual-level data cannot be shared. Researchers seeking access to CMS data for their own studies should visit the Research Data Assistance Center (ResDAC) at www.resdac.org to get started.

## Abbreviations

MVCs: Motor vehicle crashes
NJ-SHO: New Jersey Safety and Health Outcomes
Medicare FFS: Medicare Fee-for-Service
