# Supplementary material for: Linkage of Medicare insurance claims to police-reported motor vehicle crashes: advancing traffic safety research in older adult populations
Source: Inj Epidemiol. 2026 Feb 6;13:19. doi: 10.1186/s40621-026-00658-5 (PMC12973604; doi:10.1186/s40621-026-00658-5)
Supplement: Supplementary file 1 — Supplementary Material 1. [file 40621_2026_658_MOESM1_ESM.docx]

**Supplementary Table 1.** Match quality for each variable category.

| **Category** | **High** | **Medium** | **Low** | **None** |
| --- | --- | --- | --- | --- |
| Name | Full match on first and last name | Full match on first or last & partial match on the other  Partial match on both | Full/partial match on first or last & mismatch on the other | Mismatch on both first and last |
| Gender | Full | N/A | N/A | Mismatch |
| Age | Full match on birth date, either missing or at least one match on year/month/day of death. Allow for +1 on year of birth | Full match on birth date, mismatch on year/month/day of death  Partial match on both birth date and death date | Match on at least one of year/month/day of birth & year/month/day of death | Mismatch on birth date and mismatch or missing death date |
| Residence (state, ZIP) | Full match on ZIP9 | Full match on ZIP5 | Matching state  Partial ZIP match | Mismatch |

**Supplementary Table 2:** Criteria for overall match confidence assignment.

2-a. 1-to-1 Matches

| Overall match confidence (N linked in this category) | Primary patterns (percent of matches within confidence category) | Percentage with 3-4 High |
| --- | --- | --- |
| Strong (2147475) | • Name High, gender High, age High, residence High/Medium: 93.29%  • All 4 criteria High (name, gender, age, residence): 84.58% • 3 criteria High + 1 Medium (typically residence): 11.32% | 99.17% |
| Fair (446992) | • Name High, gender High, age High, residence Low/None: 84.70% • Name Medium, gender High, age High, residence Low/None: 6.99%  • Name High, gender High, age Low, residence Medium: 2.82% | 87.17% |
| Weak (10448) | • Name Low, gender High, age High, residence Low/None: 32.08%  • Name High, gender High, age Low/None: 29.55% • Key characteristics: Name and age never both High | 0% |

2.b. 1-to-Many Resolved Matches

| Overall match confidence | Primary patterns | Percentage with 3-4 High |
| --- | --- | --- |
| Strong (16560) | • All 4 criteria High (name, gender, age, residence): 88.63% • 3 criteria High + 1 Medium: 9.75% • Very similar pattern to 1-to-1 Strong matches | 99.50% |
| Fair (101298) | • Name High, gender High, age High, residence Low/None: 80.54% • Name Low, gender High, age High, residence High/Medium: 6.80% • Name High, gender High, age Medium/Low, residence Medium/Low: 5.67% | 85.03% |

Note: Weak matches were not present in 1-to-many resolved data.
